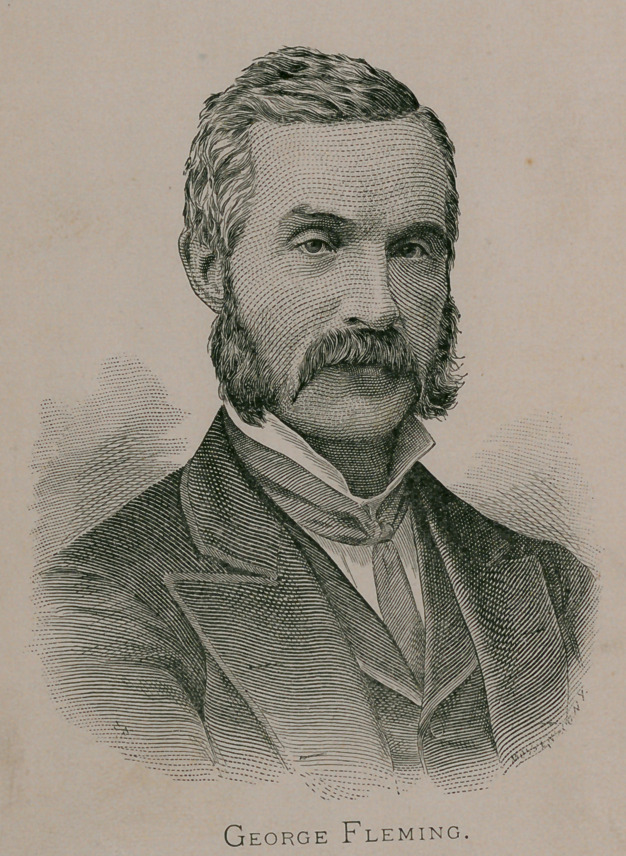# George Fleming, LL.D., F.R.C.V.S., Principal Veterinary Surgeon to the British Army

**Published:** 1886-01

**Authors:** 


GEORGE FLEMING.

the cramp, the unsteady gait, the loss of balance, &c., so marked in many cases, may be due to the nerve current from the cerebellum in its descent to the muscles finding an obstacle in the spinal cord altered by the morbid process.

### ART. V.—GEORGE FLEMING, LL.D.,F.R.C.V.S. PRINCIPAL VETERINARY SURGEON TO THE BRITISH ARMY.

Fleming was born at Glasgow, on March 11, 1833. Early in life he showed a natural taste for animals, and was of a studious turn of mind. These two qualities combined, directed him to apply his talents to veterinary science.

Professionally, he commenced life as a pupil with the late Mr. A. Lawson, with whom he worked for two years, before entering the old veterinary college, Clyde Street, Edinburgh; where he studied under the late founder of the school, Professor Dick. During his collegiate life he spent his vacations with the late Mr. John Lawson, of Manchester, in whose extensive practice he had an opportunity of observing the various details of the "Art and Science" he chose to study. Whilst a student at Clyde Street he obtained the medals for Chemistry, Materia Medica, Anatomy, Essays, the best General Examination and the Fitzwygram Prize for practical Knowledge. He qualified to practice Veterinary Medicine and Surgery by obtaining the certificate of the Highland and Agricultural Society of Scotland in 1855.

As soon as he had graduated he was offered an appointment in the Army by the late Mr. Wilkinson (then Principal Veterinary Surgeon to the Forces), which he refused, preferring to go into private practice at Burnley, in Lancashire. Six months after this Mr. Wilkinson again urged him to join the army, and he complied with the request, intending to return to his practice after the Crimean war was finished, in 1856. However he soon found that the life of a soldier was far more pleasant to his active, jocular disposition, than the humdrum and worry of a private practice ; so he made up his mind to remain in the army. In 1859 he volunteered to serve in the expedition to North China, and was present at the capture of the Taku Forts, and the different actions leading to the surrender of Pekin; he remained in China until 1861. During his stay there, he and a friend made a very hazardous journey beyond the Great Wall of China, a description of which he afterwards published in the shape of a book entitled "Travels on Horseback in Mantchu Tartary." This was the first book he wrote. In 1867 he served in Syria. He has served in the Third (King's Own) Hussars, Royal Engineers, and the Second Life Guards In 1879 he was appointed Inspecting Veterinary Surgeon at the War Office, which post he held until His Royal Highness, the Duke of Cambridge, Field Marshal, Commanding-in-Chief, offered him the appointment of Principal Veterinary Surgeon to the British Forces, in 1883, and we hope his health will be preserved so as to enable him to carry out the duties, which we are confident will in the end be advantageous to the veterinary profession and the department of which he is Principal.

Although Fleming was leading an active life as a soldier, still this did not prevent him exercising his talent for study and the advancement of his profession. He was constantly contributing articles on veterinary and the allied sciences to various periodicals,

In 1863 he read two papers before the members of the British Association, at Newcastle-on-Tyne. One on the "Geography of North China," the other on the "Ethnology of that Country."

In 1866 he presented himself for and passed the examination for the Diploma of the Royal College of Veterinary Surgeons. The following year he was elected a Vice-President of that body, when he proved to be so able and useful that in 1868 the profession elected him a member of their Council and they have re-elected him every time his office has expired. In 1880 the Council selected him to occupy the most distinguished position in the English profession, viz.: PRESIDENT OF THE ROYAL COLLEGE OF VETERINARY SURGEONS. He carried out the duties so efficiently that they kept him in office until 1884. He has also been a member of the Examining Board, for the Diploma of the R.C.V.S., since 1872.

His official duties have been no sinecure, for he has been most attentive to the welfare of the profession—watching with a keen eye the movements of those whose objects were anything but those most beneficial to the profession as a whole. He has always striven to improve the educational standard and the examinations of the student in every way. He has also strenuously endeavored to maintain the supremacy of the Royal College of Veterinary Surgeons over the colleges (teaching schools). So much has the latter been the case, that in 1875 Fleming gave up contributing to the then solitary existing veterinary periodical, and founded and edited one of his own, (The Veterinary Journal). His reasons for so doing being in our opinion ample and justifiable, as may be seen by any one who will take the trouble to refer to his editorials in the 1st number (July, 1875). The only portal by which members should be admitted as efficient to practice a profession, *in any country*, should be controlled by the profession itself, and not ruled by any section of it; and Dr. Fleming has been one of the principal guardians of this maxim in England, much to the benefit of both members and profession.

One other great work he was instrumental in completing must not be forgotten, viz.: protecting the right of using the title "VETERINARY SURGEON." Before 1881 any person, whether educated or not, could call himself a Veterinary Surgeon. Now, however no person (except those who had been practicing the veterinary art for five years previous to 1881), can use the title of "Veterinary Surgeon,"—or any other designation which would lead the public to think he was qualified—unless he had passed the examination for the Diploma of the R. C. V. S.

All this gratuitous hard work, pecuniary expenditure, and labor of love for his profession, was recognized in a very feeble but genuine manner by its members presenting him on two different occasions with testimonials. Not only has his profession acknowledged his great works, but we are pleased and gratified to know that others have done so also. Various learned continental societies have conferred their honors upon him, whilst those outside the profession have not forgotten him at home. It was only in 1883 that the University of Glasgow (his native town) recognized the veterinary profession for the first time in its history by conferring upon him their honorary degree of Doctor of Laws. In recognition of the papers he read before the British Association in 1863 Sir R. Murchison (then President of the Royal Geographical

Society) nominated him as a Fellow of that Society, and Sir Harry Parkes (then Minister to Pekin) seconded the nomination.

We could, if space and time permitted, say a great deal more; but sufficient has been done by himself to show his comrades in war of his abilities in the barracks, field and mess-room; to his professional brethren, of his untiring energies to place their profession in the position it should occupy, and which we have little doubt it soon would occupy, had we a few score of of the 3000 M.R.C.V.S. as loyal to their profession and as energetic in accomplishing its advancement as he.

We should like to say something of the social side of his character, but as we do not think this admissible in a professional journal we will simply inform our American friends that in whatever sphere we have had the pleasure of meeting him—and that is not a few—we have always found him a jolly and lively companion, earnest and true friend, and a willing, generous assistant and adviser.

## Figures and Tables

**Figure f1:**